# Single‐cell RNA‐seq of in vitro expanded cells from cranial neural crest reveals a rare odontogenic sub‐population

**DOI:** 10.1111/cpr.13598

**Published:** 2024-01-09

**Authors:** Yifan Zhao, Shubin Chen, Xiaobo Liu, Xiaoming Chen, Dandan Yang, Jiashu Zhang, Di Wu, Yanmei Zhang, Si Xie, Xiaomei Li, Zhiyuan Wang, Bo Feng, Dajiang Qin, Duanqing Pei, Yaofeng Wang, Jinglei Cai

**Affiliations:** ^1^ Innovation Centre for Advanced Interdisciplinary Medicine The Fifth Affiliated Hospital of Guangzhou Medical University Guangzhou China; ^2^ Centre for Regenerative Medicine and Health Hong Kong Institute of Science & Innovation, Chinese Academy of Sciences Hong Kong SAR China; ^3^ Bioland Laboratory, Guangzhou Regenerative Medicine and Health Guangdong Laboratory Guangzhou China; ^4^ CAS Key Laboratory of Regenerative Biology, Guangdong Provincial Key Laboratory of Stem Cell and Regenerative Medicine Guangzhou Institutes of Biomedicine and Health, Chinese Academy of Sciences Guangzhou China; ^5^ Laboratory of Cancer Precision Medicine The First Hospital of Jilin University Changchun China; ^6^ Guangdong Provincial People's Hospital Ganzhou Hospital, Ganzhou Municipal Hospital Ganzhou China; ^7^ Experimental Center of Pathogenobiology Immunology, Cytobiology and Genetics, Basic Medical College Jilin University Changchun China; ^8^ Innovation Centre for Translational Medicine The Fifth Affiliated Hospital of Guangzhou Medical University Guangzhou China; ^9^ School of Biomedical Sciences The Chinese University of Hong Kong Hong Kong SAR China; ^10^ Laboratory of Cell Fate Control, School of Life Sciences Westlake University Hangzhou China; ^11^ Guangzhou Key Laboratory of Enhanced Recovery after Abdominal Surgery The Fifth Affiliated Hospital of Guangzhou Medical University Guangzhou China; ^12^ Institute for Stem Cell and Regeneration, Chinese Academy of Sciences Beijing China

## Abstract

Ecto‐mesenchymal cells of mammalian tooth germ develops from cranial neural crest cells. These cells are recognised as a promising source for tooth development and regeneration. Despite the high heterogeneity of the neural crest, the cellular landscape of in vitro cultured cranial neural crest cells (CNCCs) for odontogenesis remains unclear. In this study, we used large‐scale single‐cell RNA sequencing to analyse the cellular landscape of in vitro cultured mouse CNCCs for odontogenesis. We revealed distinct cell trajectories from primary cells to passage 5 and identified a rare *Alx3*+/*Barx1*+ sub‐population in primary CNCCs that differentiated into two odontogenic clusters characterised by the up‐regulation of *Pax9*/*Bmp3* and *Lhx6*/*Dmp1*. We successfully induced whole tooth‐like structures containing enamel, dentin, and pulp under the mouse renal capsule using in vitro cultured cells from both cranial and trunk neural crests with induction rates of 26.7% and 22.1%, respectively. Importantly, we confirmed only cells sorted from odontogenic path can induce tooth‐like structures. Cell cycle and DNA replication genes were concomitantly upregulated in the cultured NCCs of the tooth induction groups. Our data provide valuable insights into the cell heterogeneity of in vitro cultured CNCCs and their potential as a source for tooth regeneration.

## Graphical abstract

Ecto‐mesenchymal cells of mammalian tooth germ develops from cranial neural crest cells. These cells are recognised as a promising source for tooth development and regeneration. Despite the high heterogeneity of the neural crest, the cellular landscape of in vitro cultured cranial neural crest cells (CNCCs) for odontogenesis remains unclear. In this study, we used large‐scale single‐cell RNA sequencing to analyse the cellular landscape of in vitro cultured mouse CNCCs for odontogenesis. We revealed distinct cell trajectories from primary cells to passage 5 and identified a rare *Alx3*+/*Barx1*+ sub‐population in primary CNCCs that differentiated into two odontogenic clusters characterised by the upregulation of *Pax9*/*Bmp3* and *Lhx6*/*Dmp1*. We successfully induced whole tooth‐like structures containing enamel, dentin and pulp under the mouse renal capsule using in vitro cultured cells from both cranial and trunk neural crests with induction rates of 26.7% and 22.1%, respectively. Importantly, we confirmed that only cells sorted from odontogenic path can induce tooth‐like structure. Cell cycle and DNA replication genes were concomitantly upregulated in the cultured NCCs of the tooth induction groups. Our data provide valuable insights into the cell heterogeneity of in vitro cultured CNCCs and their potential as a source for tooth regeneration.

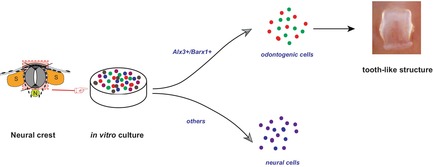

## 1 INTRODUCTION

Tooth regeneration is a promising strategy to restore the function and aesthetics of missing or damaged teeth. Tooth develops from the interactions between the oral epithelial and the underlying mesenchymal cells.^1^ Typical recombination experiments between epithelia and mesenchyme showed that the odontogenic potential (odontogenesis induction ability) shifts from epithelium to mesenchyme at bud stage (mouse embryonic days, E12 and E13).^2^, ^3^, ^4^, ^5^ Thus, scientists succeeded in using the dental epithelium before the bud stage or the dental mesenchymal component at the post‐bud stage to respectively induce other mesenchymal component or other epithelial component on tooth regeneration.^6^, ^7^, ^8^, ^9^ However, one of the major challenges is to obtain sufficient and suitable mesenchymal cells that can interact with epithelial cells to form tooth‐like structures. Neural crest cells (NCCs) are multipotent stem cells that give rise to various craniofacial tissues, including the dental mesenchyme. Therefore, NCCs are expected as promising mesenchymal cell sources for tooth repair and regeneration.

Migratory NCCs are responsible for the formation of a variety of different anatomical structures throughout the body, including nervous system, cartilage, smooth muscles, nerves, bones, dental tissues and so on.^10^, ^11^, ^12^, ^13^, ^14^ In mice, the developing notochord, along with adjacent mesenchyme induces the ectoderm on its surface to form neural plate at around embryonic days 6 and 7 (E6 and E7), which gradually closes to form neural tube.^15^ NCCs originate between the neural tube and the surface ectoderm, form a cell band along the head and tail of the embryo, and then gradually undergo epithelial–mesenchymal transformation.^16^, ^17^, ^18^ According to the in vivo localisations, neural crest can be divided into four types: cranial, cardiac, trunk and vagal NCCs. They can migrate and differentiate under the regulation of various signalling pathways, such as TGF‐beta and Wnt signalling pathways, and eventually form different tissues and organs. It is worth mentioning that in the process of craniofacial formation, a small group of CNCCs can differentiate into dental mesenchymal cells under the influence of surrounding environment and extracellular signals.^19^, ^20^ Therefore, cranial neural crest is recognised as the promising cell resource for tooth regeneration in vitro.

To date, scientists have successfully differentiated NCCs into odontoblasts, dentin, or cementoblasts in vitro within bioengineering materials or a developing tooth germ.^21^, ^22^, ^23^ Besides, pluripotent cells derived neural crest‐like cells were considered as a promising cell source for tooth regeneration.^24^, ^25^ The dental markers could be activated in the induced neural crest‐like cells derived from pluripotent stem cells by recombination with dental epithelium or in bioengineering materials. However, no successful regeneration of whole tooth with structures of dental pulp, dentin, and more importantly the enamel derived from neural crest‐interacting non‐odontogenic epithelium (other than E10.5–E12.5 dental epithelium) from neural crest‐like cells has been reported yet. Meanwhile, scientists found that the dermal denticles on the back of cartilaginous fish have similar structures of enamel, dentin and pulp in teeth.^26^ Dermal denticles on the back are proved to be derived from the migration and differentiation of trunk neural crest (TNC), indicating the trunk neural crest cells (TNCCs) may have the odontogenesis potential as those from the cranial neural crest.

The NCCs become highly heterogeneous during the migration and differentiation.^27^ The dorsal neural tube, representing the origin area of NCCs, consists of several sub‐populations with heterogeneous expression patterns containing neural and neural crest markers, pluripotency factors, differentiation markers and genes associated with cell proliferation or cell death. Recently, scientists have applied single‐cell RNA sequencing to demonstrate the notable heterogeneity within the CNCC and TNCC in vivo, and their cell fate transitions during the embryonic development.^28^, ^29^ However, the cellular heterogeneity of in vitro cultured CNCCs as the cell resource for tooth induction remains unclear. In this study, we performed large‐scale single‐cell RNA sequencing (~59,000 cells) to demonstrate the cell trajectories of in vitro cultured and expanded mouse CNC cells from primary cells to passage 5. A rare sub‐population in primary CNCCs is identified to differentiate from odontogenic clusters. Further, we applied SENIC analysis to identify the key factors to drive the odontogenic cell differentiation. Moreover, combined with post‐bud dental epithelium, for the first time, we successfully induced the whole tooth‐like structures in mouse kidney using in vitro cultured cells from both CNC and TNC. Cell cycle, DNA replication, and Wnt signalling pathway genes are upregulated for successful tooth induction. These findings might contribute to further studies of NCCs as a promising cell resource for tooth repair/regeneration.

## 2 RESULTS

### 2.1 Single‐cell RNA sequencing revealed the heterogeneities of primary CNCCs and identified a rare *Alx3*+/*Barx1*+ sub‐population

**FIGURE 1:**
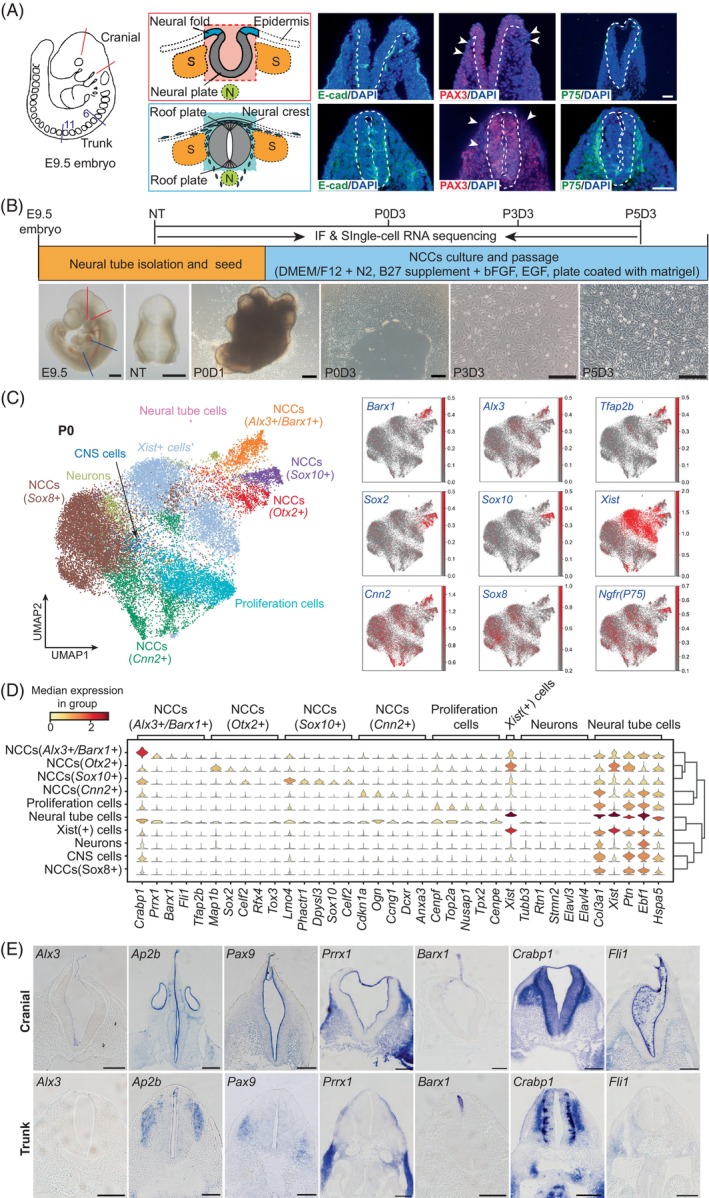
Isolation and identification of primary neural crest cells. (A) Left, cranial and trunk neural tubes in the embryo; Middle, schematic diagram of the reserved part of the neural tube, and the part outside the red frame or blue frame is removed; Right, immunofluorescence staining of neural tube and surrounding tissues with E‐cadherin, Pax3 and p75. DAPI (4′,6‐diamidino‐2‐phenylindole) was used for nuclear staining. Scale bar: 100 μm. (B) Flow chart of NCCs isolation and culture, including schematic diagram of sampling points for immunofluorescence and single‐cell RNA sequencing. Scale bar: embryo and neural tube 500 μm, cell culture 200 μm. (C) UMAP visualisations of single‐cell RNA‐seq analysis of primary CNCCs. (D) Violin plots of top marker genes of each cell type of CNCCs primary cells. (E) Expression patterns of *Alx3*, *Ap2b*, *Pax9*, *Prrx1*, *Barx1*, *Crabp1* and *Fli1* in E9.5 mouse cranial and trunk neural tube. Scale bars correspond to 200 μm. CNCC, cranial neural crest cell; NCC, neural crest cell.

To investigate the heterogeneity of Primary CNCCs at the single‐cell transcriptome level, the neural tube in mouse E9.5 embryos was dissected from the distal head between two red lines (Figure 1A, left), followed by removal of the surrounding tissues to keep the area of the light red square only (Figure 1A, middle). At E9.5, the TNC develops to a more mature state than the CNC, showing fusion of the neural folds on both sides with the appearance of closed neural tube and roof plate in the trunk part. However, the fusion of the neural folds just starts with the existence of neural groove in the cranial part (Figure 1A, middle and right). We isolated the TNCCs as well from the distal back which are denoted as ganglion 6–11 and showed between two blue lines in Figure 1A. The area of light blue square in TNC was prepared for further cell culturing (Figure 1A, middle, Figure S1A). Epithelial surface marker E‐cad, neural progenitor cell marker PAX3 and NCC markers P75 were selected for immunofluorescence to suggest the in vivo localisations of the isolated neural tube (Figure 1A, right). As expected, E‐cad‐positive cells could be generally detected in almost all cells of the dissected region of CNC and TNC, displaying in both neural tubes/folds/grooves and its surrounding region. Positive PAX3 expression is present in most cells and absent in a small number of cells around the neural folds (arrowheads) with surrounding thin layers in the CNC and roof plate (arrowheads) in the TNC. In particular, most P75 positive cells are found to slightly display surrounding the neural tube in CNC and widely spread surrounding the neural tube in TNC. These data indicate that the NCCs are migrating and undergoing the epithelial–mesenchymal transition (EMT), which confirms that migrating NCCs are isolated and cultured in vitro with neural tubes/folds in our study. With explant‐attached method, we cultured and passaged the cells on Matrigel‐coated plates with a slightly modified culture medium from the previous report,^30^ containing N2, B27, bFGF, EGF in DMEM/F12 based medium (Figure 1B) (detailed in Section 4.2). In detail, cells climbed out from the cultured neural tubes at day 1 of passage 0 (P0D1). The morphologies of those cells implied their heterogeneities; some cells were spindle‐shaped, while some cells were irregular quadrilateral. Neural tube and miscellaneous cells were removed at P0D3, and then the fully proliferated primary cells at P0D6 were passaged and cultured until P5. At P3, the cells showed a variety of shapes, including irregular quadrilateral, long spindle, triangle and circular. Besides, the cultured cells further displayed two types clearly, spindle‐shaped or ellipse‐shaped at P5. In addition, a small amount of nerve filaments (arrowheads) could be observed in some spindle‐shaped cells (Figure 1B, Figure S1B), indicating part of cells at P5 differentiate towards neural lineages.

To further identify the heterogeneity of primary CNCCs, we performed single‐cell RNA sequencing (scRNA‐seq) of P0 cells using 10× genomics platform. A total number of 5569 cells were detected. Through unsupervised clustering (Leiden algorithm) and marker analysis, we identified 10 cell types within P0 primary CNCCs, including *Cnn*2^+^ migratory NCCs, *Sox8* ^+^ migratory NCCs, *Sox10* ^+^ NCCs, *Alx3* ^+^/*Barx*1^+^ NCCs, *Otx2* ^+^ NCCs, proliferation cells, *Xist* ^+^ cells, central nervous system (CNS) cells, neurons and a few residual neural tube cells (Figure 1C, left). P75 was highly co‐expressed with *Sox8*, suggesting the post‐migratory state of the primary CNCCs in culture (Figure 1C, right). Moreover, *Sox2* was highly expressed in *Sox10* ^+^ and *Otx2* ^+^ NCC groups, which implied the primary CNCCs had initiated the differentiation toward neural lineage at P0 (Figure 1C, right). Our data reveal the remarkable complexity and heterogeneity among the primary CNCCs.

Strikingly, a rare sub‐population of *Alx3* ^+^/*Barx1* ^+^ cells was identified in P0 primary CNCCs (Figure 1C left for cell type clustering, Figure 1C right for the gene expression levels of *Alx3* and *Barx1*, respectively). *Crabp1*, *Tfap2b*, *Prrx1* and *Fli1* were also specifically expressed in this subset of cells (Figure 1D). According to our previous study, *Alx3* is the critical transcriptional factor in incisor mesenchyme, while *Barx1* and *Tfap2b* are crucial in molar mesenchyme.^31^ Moreover, *Crabp1* and *Fli1* were previously reported to be expressed in dental papilla.^32^, ^33^ Furthermore, in situ expression of *Alx3* was observed in cranial neural crest, while six genes (*Ap2b*, *Pax9*, *Prrx1*, *Barx1*, *Crabp1* and *Fli1*) were observed in both CNC and TNC (Figure 1E).

Overall, our analysis highlights a rare cell sub‐population in primary CNCCs with the potential of odontogenesis.

### 2.2 In vitro cultured CNCCs divided into distinct differentiation trajectories during passages and led to two odontogenic sub‐populations

**FIGURE 2:**
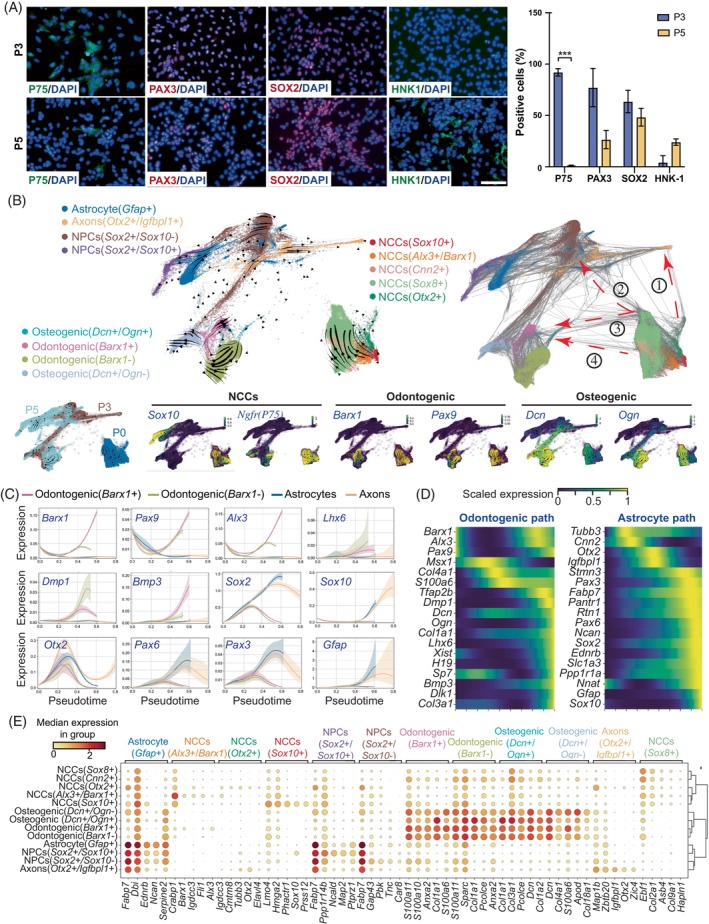
Single‐cell landscape of cultured CNCCs lineage specifications in vitro. (A) Left: Characterisation of P3 and P5 CNCCs with immunofluorescence. Scale bar corresponds to 100 μm. Right: Bar plots of positive cells P3 and P5 CNCCs with immunofluorescence as statistical results. (B) Single‐cell RNA‐seq analysis of in vitro cultured CNCCs in P0, P3, and P5: (top) Heterogeneous cell types and landscape of in vitro cultured CNCCs; (bottom) markers gene expressions and velocity in each lineage. (C) Trends of marker genes expressed in specific lineages as pseudo time. (D) Heat map showing the marker genes expressed in odontogenic path and astrocyte path. (E) Dot plots of top marker genes of each cell type in CNCCs differentiation landscapes. CNCC, cranial neural crest cell.

Detected by immunofluorescence (Figure 2A, left), NCC marker P75 were downregulated gradually from P3 to P5 during the passaging of primary CNCCs in vitro. It was speculated that the cells began to differentiate. Meanwhile, neural progenitor cell (NPC) markers *Sox2/Pax3* were down regulated, while CNS cell marker *Hnk1* in mice was upregulated (Figure 2A, right). Those results suggested the cell fates of primary CNCCs transited from the neural crest to neural progenitor/CNS during in vitro passages. Still, ~50% of P5 cells were *Sox2* negative, which indicated other cell lineages during the differentiations.

To demonstrate the lineage specifications of primary CNCCs during in vitro culture and passaging, P3 and P5 cells were collected and scRNA‐seq was performed, respectively. Together with P0 cells, 58,493 cells in total were detected to draw the single‐cell resolution cell landscape of CNC lineage specifications in vitro. Clustering and marker analysis of the transcriptomes of P0, P3 and P5 cells revealed the expansion of a variety of cell types over the differentiation time. We classified the cells into four neural cell types (*Gfap* ^+^ astrocyte, *Otx2* ^+^/*Igfbp1* ^+^ axons, *Sox2* ^+^/*Sox10* ^−^ NPCs and *Sox2* ^+^/*Sox10* ^+^ NPCs) and four osteogenic/odontogenic cell types (*Dcn* ^+^/*Ogn* ^−^ osteogenic, *Dcn* ^+^/*Ogn* ^+^ osteogenic, *Barx1*+ odontogenic and *Barx1*‐ odontogenic) at P3 and P5 stages (UMAP illustrations in Figure S1C,D). *Sox2* ^+^/*Sox10* ^−^ NPCs were only observed in P5 but not in P3. Odontogenic cells were identified according to the expression level of *Pax9* marker. We also examined the expression patterns of key genes for neural (*Sox2*, *Sox10*, *Pax3*, *Pax6*, *Gfap* and *Igfbpl1*) and osteogenic/odontogenic (*Ogn*, *Dcn*, *Bmp2*, *Pax9*, *Barx1* and *Dmp1*) differentiation at P3 and P5 stages (Figure S1E,F). These results revealed two distinct cell differentiation trajectories: neural lineage and osteogenic/odontogenic lineage.

To better investigate the multilineage differentiation landscape of primary CNCCs in vitro, we applied RNA velocity analysis to recover the directed dynamic information by leveraging splicing kinetics of in vitro cultured CNCCs combined P0, P3 and P5 (Figure 2B, top left). The predicted future state of cells was visualised based on their gene expression profiles using arrows in the FA embedding (Force Atlas 2 algorithm). The arrows indicated the direction and magnitude of the transcriptional changes in each cell. The arrows were computed from the ratio of unspliced and spliced mRNA abundances, which represented the rate of change of each gene.^34^ The figure revealed the transcriptional dynamics of NCCs along the differentiation trajectories, as well as the potential fate of intermediate cell states. From five groups of NCC cells at P0 (*Sox10* ^+^, *Alx3* ^+^/*Barx1* ^+^, *Cnn2* ^+^, *Sox8* ^+^ and *Otx2* ^+^, respectively), four possible cell differentiation paths from NCC to neural or osteogenic/odontogenic lineages (Figure 2B, top right): ①: *Otx2* ^+^ NCCs could lead to *Otx2* ^+^/*Igfbpl1* ^+^ axons; ② and ③: *Sox8* ^+^ NCCs could lead to *Sox2* ^+^/*Sox10* ^−^ NPC and osteogenic cells, respectively; ④: *Alx3* ^+^/*Barx1* ^+^ NCCs could lead to *Barx1* ^+^ odontogenic cells. Based on the dynamics of cell differentiation paths, the genes associated with lineage were clearly classified (Figure 2B bottom, Figure S2A). Furthermore, we performed pseudo‐time analysis to draw the gene expression levels in four cell differentiation paths (Figure 2C): odontogenic genes, *Barx1*, *Pax9* and *Alx3*, were expressed but low in early stage, and subsequently high upregulated in *Barx1* ^+^ odontogenic path. Those factors are critical in E12.5–E13.5 dental mesenchymal cells, according to our previous report.^31^ *Bmp3* was silenced at the early stage and activated in *Barx1* ^+^ odontogenic cells. Moreover, *Lhx6* and *Dmp1* were silenced at the early stage and expressed in *Barx1* ^−^ odontogenic cells. Collagen genes (*Col1a1*, *Col3a1* and *Col4a1*) and osteogenic genes (*Xist*, *Dcn*, *H19* and *Ogn*) were also activated in odontogenic paths (illustrated as heat map in Figure 2D, and UMAP visualisations in Figure S2A). On the other hand, neural markers were dynamic during the multilineage landscape in neural paths. Astrocyte genes were activated in the late stage of the cell differentiations (Figure 2D, Figure S2A). Besides, novel genes specifically expressed in each cell type were shown in our scRNA‐seq analysis (Figure 2E).

We further applied SCENIC analysis to gain insight into the gene regulatory networks (GRNs) and inferred critical transcription factors (TFs) for odontogenic differentiation of in vitro cultured CNCCs. SCENIC can predict TFs, jointly named as regulons, through co‐expression patterns and cis‐regulatory motif analysis, along with their candidate target genes.^35^ Two GRNs, *Prrx1* and *Trap2b*, were identified to drive the differentiation lineage specifically from *Alx3* ^+^/*Barx1* ^+^ NCCs to *Barx1* ^+^ odontogenic cells (Figure S2B,C). There were 32 common genes between *Prrx1*‐GRN and *Tfap2b*‐GRN, which related to Wnt signalling pathway and osteoblast differentiation according to gene ontology analysis. We analysed the expression dynamics of the potential targets of *Prrx1*‐GRN and *Trap2b*‐GRN during the NCCs to odontogenic cell transition. We used a heat map to visualise the scaled expression levels of these genes in the *Barx1*+ lineage along the pseudo‐time (Figure S2D). The downstream genes of *Prrx1*‐GRN were mostly active at the late stage and were involved in biological processes related to bone formation and tooth development. These genes were enriched for specific gene ontology (GO) terms in the Receptor Tyrosine Kinases (RSK) signalling pathway, ossification and odontogenesis (Figure S2C, right). The genes that were downstream of *Trap2b*‐GRN were mostly active at the intermediate stage and were involved in biological processes related to tissue specification and growth. These genes were enriched for specific GO terms in cell fate commitment, epithelial cell proliferation and myofibril assembly (Figure S2C, right).

In summary, there are two distinct developmental trajectories during CNC in vitro passages. Eventually, the cells divided into neuro‐lineage and osteo/odonto‐lineage in P3 and P5. Two odontogenic cell subgroups were identified during the in vitro culture and expansion.

### 2.3 NCCs cultured from both cranial and trunk neural tubes exhibited the tooth regeneration potential

**FIGURE 3:**
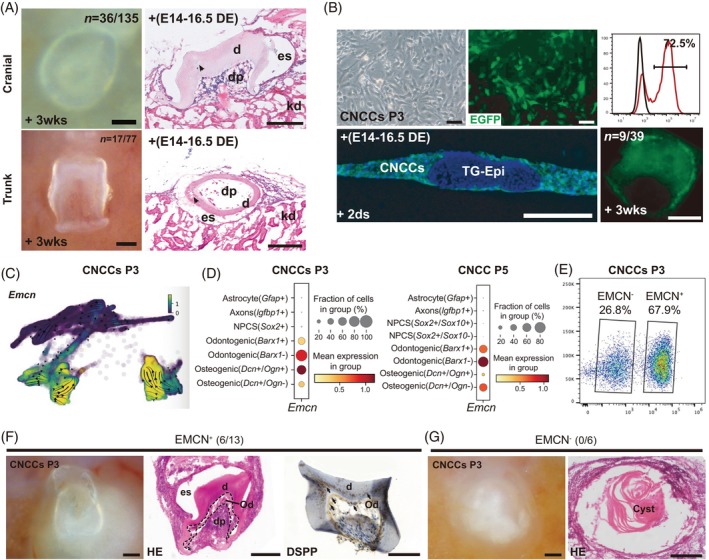
Characterisation of NCCs as seed for tooth regeneration from cranial (upper row) and trunk neural tube (bottom row). (A) Left, regenerated tooth‐like structures from the recombinants of NCCs and E14.5 DE after transplantation under renal capsule for 3 weeks. Scale bar corresponds to 100 μm. Right, H&E staining of relative tooth sections showing the tooth‐like structures containing dental pulp (dp), dentin (d), enamel space (es) and kidney (kd). Scale bar corresponds to 200 μm. (B) Upper row, EGFP labelled neural crest cells and labelling efficiency detected by flow cytometry. Lower row, sections of the regenerated tooth germ constructed by EGFP NCCs and results of tooth formation. Scale bar corresponds to 200 μm. (C) scRNA‐seq analysis of EMCN expression in cells derived from NCCs. (D) Dot plots to show the expression levels of *Emcn* in each cell groups in P3 and P5. (E) Proportions of EMCN+ and EMCN− cells sorted at P3. (F and G) Tooth inductions with EMCN+ and EMCN− cells sorted from P3; dental pulp (dp), dentin (d), enamel space (es) and bone (B); Scale bar: 100 μm. DE, dental epithelium; H&E, haematoxylin and eosin; NCC, neural crest cell.

It was demonstrated that TNCCs do give rise to odontoblasts of trunk dermal denticles in cartilaginous fish models by cell lineage tracing. This cell population with the likely primitive skeletogenic potential was pinpointed to be a neural crest origin of dentine throughout the ancestral vertebrate dermal skeleton. Thus, we also isolated NCCs from the trunk part at somite 6th to 11th in E9.5 mouse embryos (Figure 1A, tissues between blue lines in left). The cells isolated from the trunk part exhibit similar cell shape to those of the cranial part during the passages (Figure S1A). To confirm whether these cultured cells from both CNCCs and TNCCs could induce tooth regeneration, we pelleted cells randomly harvested from P3, P4 or P5 by centrifugation, respectively, and then each cell pellet was recombined with a piece of epithelium from E14.5 to E16.5 mouse tooth germ, followed by transplantation under the mouse renal capsule. After 3 weeks, we observed tooth‐like structures in the kidney from both cranial and trunk groups (Figure 3A). The induction rate of tooth formation is 26.7% (*N* = 36/135) and 22.1% (*N* = 17/77) in the cranial and trunk groups, respectively. The sizes of most tooth‐like structures in both groups are smaller than those derived from tooth germs transplanted after 3 weeks. After the decalcification, confirmed by haematoxylin and eosin (H&E) staining in sections, the tooth‐like structures contain dental pulp (dp), dentin (d) and enamel space (es) in sections. Nevertheless, the cells in the regenerated structures look unhealthy with loose arrangement, including the dental pulp cells, surrounding tissues and kidney tissues of host (kd).In particular, some irregular structures were detected in the local areas of the regenerated dentin (Figure 3A, arrowheads).

To confirm the origin of the mesenchymal component, we used lentivirus system to encode enhanced green fluorescent protein (EGFP) with the cultured cells from CNCs at P3 or P5 (Figure 3B). The EGFP infection rate is 72.5%. All mesenchymal cells in the 2‐day recombinant germs are EGFP positive, indicating the odontogenesis induction is from the cultured NCCs. As expected, the rate of tooth formation in GFP signal‐containing group is 23.1% (*n* = 9/39), which represents the mesenchymal‐derived dentin, dental pulp, and the surrounding bone tissues.

### 2.4 Cells from odontogenic path can induce tooth‐like structures

Next, we identified a cell surface marker based on scRNA‐seq data, EMCN, which is specifically expressed in the odontogenic path in P3 and P5 (Figure 3C). We also drew the dot plots from scRNA data to show *Emcn* was expressed in odontogenic and osteogenic cells but not in neural cells, in both P3 and P5 (Figure 3D). We sorted the P3 cells into EMCN+ and EMCN‐ groups and performed tooth inductions. The EMCN+ and EMCN− cells accounted for 67.9% and 26.8% of the total P3 cells, respectively (Figure 3E). The EMCN+ cells showed a ~50% success rate of tooth induction (6/13, Figure 3F), while the EMCN− cells failed to induce any tooth‐like structures (0/6, Figure 3G). The success rate was increased. Moreover, the tooth‐like structures derived from EMCN+ cells contained dental pulp (dp), dentin (d) and enamel space (es), as confirmed by H&E staining (Figure 3F, left and middle), whereas the EMCN− cells only produced the cyst (Figure 3G). Importantly, the tooth‐like structures show much better structures in each part, such as the dentin (d) and the odontoblast layer (od) derived from EMCN+ sorted cells, which displays DSPP positive reaction by immunohistochemistry (Figure 3F right, arrows). This result indicates a relatively pure cell population would greatly contribute to healthy tooth formation.

### 2.5 Cell cycle and DNA replication genes are critical in tooth induction

**FIGURE 4:**
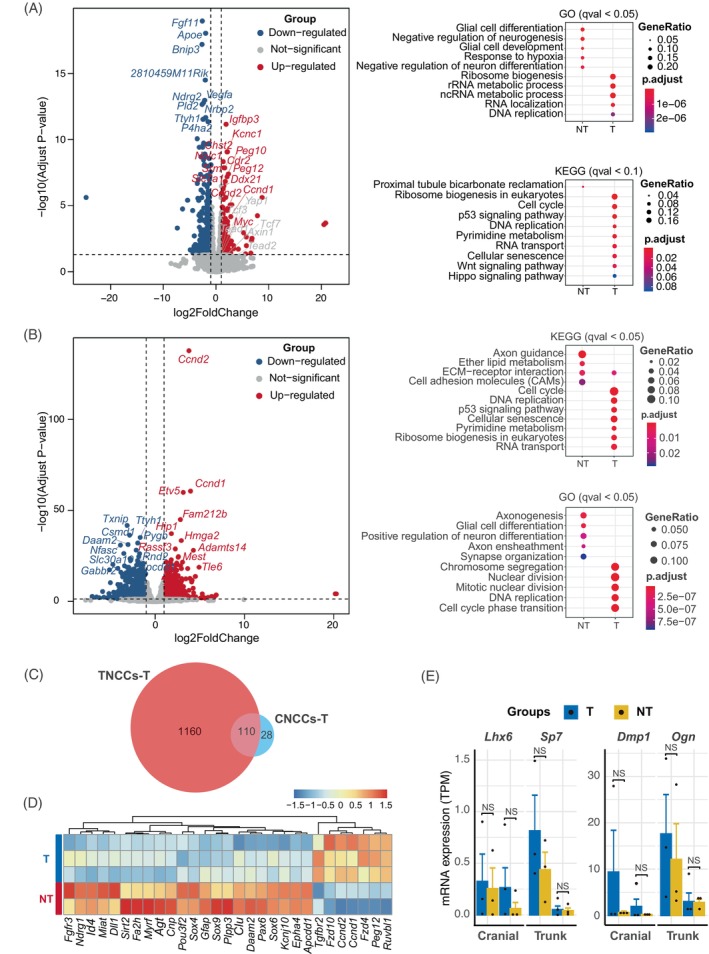
RNA‐seq analysis and differentiated expressed genes (DEGs) of CNCs and TNCCs which succeeded or failed to induce the whole tooth‐like structures (T vs. NT). (A and B) Volcano plots (left) and gene ontology analysis (right) to show the DEGs of CNCCs (A) or TNCCs (B) which succeeded or failed to induce the whole tooth‐like structures (T vs. NT). (C) Venn diagram of genes upregulated in CNCCs and TNCCs which succeed in inducing the whole tooth‐like structures. (D) Heat map of the expression patterns of the most significant DEGs. (E) Bar plots of the expression levels of Lhx6, Sp7, Dmp1 and Dgn between T and NT groups from bulk RNA‐seq. CNC, cranial neural crest; CNCC, cranial neural crest cell; TNCC, trunk neural crest cell.

To investigate the critical genes in tooth formation, we performed RNA‐seq experiments of CNC in vitro cultured cells and analysed the differential expressed genes between success and fail groups in tooth formation (T vs. NT) (Figure 4A,B). Cell cycle genes *Ccnd1/2* were both upregulated in CNCC and TNCC groups. In addition, GO analysis suggested genes involved in DNA replication, RNA metabolism, as well as cell cycling/nuclear divisions are critical for the tooth formation. Moreover, we observed 138 genes overexpressed in the successful tooth formation group of CNCC sample and 1270 genes in TNCC sample, with 110 common genes (Figure 4C). *Ccnd1/2*, *Tgfbr2*, *Fzd4/10*, *Peg12* and *Ruvbl1* are the respective genes of T groups (Figure 4D). These genes are receptors of Wnt signalling pathway or Wnt target genes. On the other hand, neural genes *Sox9*, *Gfap* and *Pax6*, which could lead to glial cell development and axongenesis, were upregulated in NT groups. Therefore, our result suggested cell cycle and DNA replication genes *Ccnd1/2* and Wnt signalling pathway are crucial for the tooth formation led by in vitro expanded NCC cells.

We further check the expression levels of odontogenic gene markers identified from scRNA data, and the expression of odontogenic markers between T and NT groups. Among those markers, only *Lhx6*, *Sp7*, *Dmp1* and *Ogn* showed the differences between T and NT groups with no statistical significance (Figure 3E). Furthermore, we found that the genes downregulated in T group were mainly related to neural development, while the genes upregulated in T group were associated with cell cycle, nuclear division and DNA replication processes. These findings suggest that the optimal cell proliferation state with appropriate levels of odontogenic markers is the key factor for successful tooth induction in kidney experiments.

## 3 DISCUSSION

Tooth organ develops from the interaction between the oral epithelia cells and the dental mesenchymal cells originating from the cranial neural crest. Therefore, NCCs are expected as promising mesenchymal cell sources for tooth repair and regeneration. The amount of post‐migratory CNCCs is limited when isolated from neural tube. Besides, CNC differentiation in vitro is highly dynamic. Scientists have been facing the challenge of inducing the odontogenic lineage efficiently from neural crest cell culture and expansion for years. Therefore, it is essential to understand the heterogeneity of in vitro cultured NCCs in high resolution and identify the odontogenic sub‐population and differentiation landscape, which can interact with epithelium to form the whole tooth‐like structures. scRNA‐seq is a powerful technology for resolving heterogeneous cell types and provides insights into cell functional heterogeneity. In this study, we demonstrated the heterogeneity and differentiation potential of in vitro cultured CNCCs for odontogenesis by scRNA‐seq analysis. The key finding from our result is the identification of odontogenic differentiation landscape of in vitro cultured and expanded CNCCs in single‐cell resolution. A rare *Alx3* ^+^/*Barx1* ^+^ cell sub‐population of primary CNCCs can differentiate into two odontogenic cell clusters (*Barx1+* vs. *Barx1‐ cells*) at P3 and P5. Our results suggest that EMCN is a marker of cells from the odontogenic path.

The in vivo development of CNCCs has been profiled and reported previously.^29^ Moreover, our previous study^31^ and another recent study,^36^ which examined the scRNA profiles of molar dental pulp, provided the cell landscape and regulatory atlas during the dental pulp development. Although neural and osteogenic/odontogenic cell lineages have been observed in both in vivo and in vitro cell differentiation, the in vitro cell lineages are less heterogeneous than the in vivo development. This may be due to our efforts to optimise the culture medium to enrich the odontogenic cells. For the rare cell sub‐populations in other lineages during in vivo CNCC development, they may not survive in our medium. The researchers need to develop their own medium to expand the relevant cell sub‐populations for other applications.

Another important finding is we succeeded to form the whole tooth‐like structure using both CNCCs and TNCCs in vitro. Although a previous publication suggested that teeth could be formed in combinations of fresh dissected CNC (*n* = 36/117) or TNC (*n* = 5/40) at E8 with mandibular arch epithelium at E9 and E10,^19^ it is ambiguous that mandibular arch epithelium at early stage are odontogenic thus can induce non‐odontogenic mesenchyme to form the teeth.^2^ In the same article, the authors also reported that these freshly dissected neural crests from both origins could not produce teeth (CNC, *n* = 0/18; TNC, *n* = 0/5) when recombined with limb epithelium (E9–E11). In our study, we observed the tooth‐like structures including dentin and dental pulp developed from cultured NCCs. The cells can interact with non‐odontogenic post‐bud dental epithelium to induce enamel formation in the tooth‐like structures in 3 weeks. These results verify our hypothesis that TNCCs can induce the tooth regeneration, although they do not develop into craniofacial in vivo. More importantly, much better structures with distinct odontoblast layers were displayed in the regenerated teeth when the cultured NCCs were sorted by a new marker EMCN of the osteogenic/odontogenic lineage. In addition, our RNA‐seq analysis suggested the upregulation of cell cycle, proliferation, and Wnt signalling pathway genes is the signature of NCCs in vitro which can successfully induce the tooth regeneration. It provides extra valuable basis for the identification of promising cell resources for tooth repair/regeneration besides odontogenic markers.

A limitation of this study is the current odontogenic induction protocol is not robust. Due to the high heterogeneity of in vitro cultured NCCs, the induction rate of whole tooth‐like structures is varied between batches, while the induced tissues in the regenerated teeth are not under a perfect condition as well. However, scRNA‐seq still provides significant clues on deliberate activation of Wnt signalling pathway and collagens. Encouragingly, several research groups including us, are demonstrating the molecular mechanism of Wnt signalling pathway and epigenetic regulations. Besides, functional study of critical factors to drive the odontogenic differentiation, besides bioinformatic predictions, will be conducted in future research.

Moreover, our scRNA‐seq data revealed some intriguing findings. One of them is the expression pattern of *Msx1*, a crucial gene for dental mesenchyme and pulp development. We found that *Msx1* was only expressed in the intermediate stage of odontogenic differentiation path in CNCC in vitro culture, but not in the final stage, as shown in Figure 2D. This indicates that *Msx1* may regulate other odontogenic genes as a trigger in temporal manner. Another finding is the identification of two odontogenic *Barx1*+ and *Barx1*− cell populations, which likely correspond to incisor and molar tooth germs, respectively. *Lhx6* and *Dmp1* are activated during the differentiation of *Barx1−* cells (Figure 2C). These may be early gene expression patterns for tooth type specification, and their roles in odontogenesis warrant further investigation.

In conclusion, our study herein provides the evidences that a rare *Alx3+*/*Barx1+* sub‐population in primary CNCCs was identified. They differentiate into two odontogenic clusters characterised by *Barx1+* and *Barx1*− cells, respectively. Moreover, the whole tooth‐like structures were induced from both CNC and TNC. Our study presents the first successful case of whole tooth‐like structure formation from NCCs cultured and expanded in vitro, which provides a valuable insight into using NCCs as the resource in tooth regeneration.

## 4 MATERIALS AND METHODS

### 4.1 Animal ethics

This animal experimental study was approved by the committee on the Ethics of Animal Experiments and Human Subject Research of Guangzhou Institutes of Biomedicine and Health (Guangzhou, China) with laboratory animal welfare and ethics code N2022075. All of the protocols were performed under the consent of the committee in this study.

### 4.2 Cell isolation and cell culture

The CNCC and TNCC were separated from embyonic day 9.5 (E9.5) ICR mice. The pregnant ICR mice were sacrificed and the neural grooves or neural tubes of the embryos were separated. The neural grooves or neural tubes with surrounding thin layers of tissue were dissected from the cranial and trunk parts, respectively (Figure 1A). All the dissected specimens were collected and digested in Dispase (1.5 mg/mL, Gibco, 17105‐041) at 37ºC for 10 min. After digestion, the specimens were washed three times gently with DMEM/F12 (Hyclone, SH30023.01) and seeded on Matrigel (Corning, 354277) coated six‐well plate. Samples were incubated in the culture medium containing DMEM/F12 supplemented with 200× N2 supplement (Gibco, 17502‐048), 100× B27 supplement (Gibco, 17504‐044), 20 ng/μL bFGF and 20 ng/μL EGF. After incubation at 37ºC and 5% CO_2_ for 48 h, the rest tissue blocks were picked up and the media in the wells were changed every two days. The cultured cells were passaged by a dilution of 1:3 using accutase (A6964, Sigma‐Aldrich) once every 3 or 4 days. For scRNA‐seq, the primary tissues were seeded on Matrigel‐coated 24‐well plate one by one. Each sample was incubated in a single well with 200 μL culture medium, and followed by adding 300 μL culture medium lightly into each well after incubation at 37ºC and 5% CO_2_ for 24 h. The following steps were the same as above.

For single‐cell RNA sequencing, six embryos at E9.5 were isolated for each sample on cranial neural crest. For bulk RNA sequencing of three samples, three biological replicates were performed for success and fail groups in tooth formation (T vs. NT) in both CNC and TNC.

### 4.3 Single‐cell preparation

The cells isolated from the cranial part in a 24‐well plate as P0. For single‐cell preparation, the single‐cell suspension from cells at P0, P3 and P5 was respectively prepared by treating with accutase for 6 min at 37°C. Cells were then pelleted and washed with 1× Dulbecco's phosphate buffered saline solution (DPBS) containing 0.04% BSA two times to remove ambient RNA as well as minimise cell aggregation. Cells were filtered using a 70‐μm cell strainer to remove the remaining cell debris and large clumps.

### 4.4 Single‐cell RNA sequencing

Cell concentration was determined using a haemocytometer and adjusted to obtain the target concentration for the 10× Chromium chip loading. The cells were loaded into the 10× Chromium Controller, and the Single Cell 3′ Reagent Kit v2 was applied according to the manufacturer's protocol. Following library preparation and quantitation, the libraries were sequenced on the Illumina NextSeq 500 platform. The sequencing of libraries was performed by Annoroad Gene Technology Co., Ltd, Beijing, China.

### 4.5 Single‐cell RNA‐seq data analysis

scRNA‐seq data were pre‐processed using 10× genomics cellranger pipeline. Briefly, reads were aligned to the mouse genome (mm10) with the setting ‘‐‐r1‐length = 26 ‐‐r2‐length = 98’. Other default cellranger parameters for 10× genomics were used to get the UMI count matrix. Downstream analysis was performed by SCANPY.^37^ The UMI count matrix was lightly filtered to exclude cell barcodes with low numbers of counts. Cells with less than 200 genes detected or more than 5% fraction of mitochondrial or beta‐globin counts were removed. Genes with less than three cells detected were removed. The genes with fold change 2.0 and adjusted *p* value <0.05 (Wilcoxon test) were considered to be differentially expressed. The GO analysis was performed by clusterProfiler.^38^ GRN analysis was performed by pySCENIC.^35^ Data visualisations were performed by Seurat^39^ or SCANPY.^37^ RNA velocity analysis^40^ was performed in scVelo^41^ and dynamo^34^ packages in python.

### 4.6 Whole‐mount in situ hybridisation

We collected the mouse embryos at E9.5 and fixed them with 4% paraformaldehyde (PFA) in phosphate buffereed saline solution (PBS). We then dehydrated all samples for whole‐mount in situ hybridisation (WISH) in gradient methanol (MeOH) and stocked them in 100% MeOH at −20°C for further processing. We washed the whole specimens completely in PBS with 0.1% Tween20 (PBST) and incubated them with proteinase K (20 g/mL in PBST) for 4 min. After washing in PBST, we refixed the samples in 4% PFA + 0.25% glutaraldehyde for 20 min, followed by prehybridisation at 70°C for 2 h in a hybridisation buffer containing 50% formamide. We maintained hybridisation overnight at 70°C in a hybridisation buffer with 0.2–0.5 g/mL of the riboprobe. We continuously washed the samples gradient at 70°C to remove the residual probe in a 2× standard saline citrate (SSC) twice, 2 × SSC + 0.1% CHAPS twice, 0.2 × SSC + 0.1% CHAPS once, 0.1× SSC + 0.1% CHAPS once and finally TBT twice. We then incubated the tissues for 3 h with 15% FBS in TBT for blocking nonspecific binding. Next, we incubated the tissues with anti‐digoxigenin antibodies, diluted to 1:5000 in blocking solution overnight. We washed the residual antibodies in TBT and equilibrated the tissues in a colour buffer, including 100 mmol/L Tris (pH 9.5), 50 mmol/L MgCl_2_, 100 mmol/L NaCl and 0.1% Tween 20.

The hybridisation probe used in this study was transcribed from pGSI plasmids in vitro, which inserted partial CDS fragments of target genes. Except *Barx1*, other plasmids used to generate probes were synthesised with the plasmid maps by Guangzhou IGE biotechnology. T7 polymerase was used for antisense mRNA transcriptions. After in situ hybridisation, the specimens were cryosectioned with the cross sections of embryo body at a thickness of 16 μm.

*Barx1*:

Forward 5′‐CGAAAGCCAAGAAAGGACG‐3′.

Reverse 5′‐ACCGAAATTGCGAGGACTGA‐3′.

### 4.7 Immunofluorescence

Before Immunofluorescence staining, the cells were fixed with 4% PFA in PBS. After several washes with 0.01 M PBS for the cells and frozen sections, the cultures were further incubated with the primary antibodies in PBS plus 5% BSA, 0.1% Triton X‐100 overnight at 4ºC. Primary antibodies were p75NTR (Abcam, ab8875) diluted 1:500, HNK1 (Sigma‐Aldrich, C6680) diluted 1:1000, PAX3 (Abcam, ab180754) diluted 1:300, Sox2 (Abcam, ab97959) diluted 1:1000 and E‐cadherin (CST, 24E10) diluted 1:200, and were visualised with species‐specific secondary antibody conjugated to the fluorescent labels Alexa Fluor® 488 or 568 (1:500; Invitrogen). The anti‐fade medium containing 4′, 6‐diamidino‐2‐phenylindole (DAPI, Sigma‐Aldrich) was used to counterstain nuclei in cells. Finally, the sections were observed using an Axio Scope A1 (Carl Zeiss, Jena, Germany) with an AxioCAM MRc5 (Zeiss), ZEN2010 confocal microscopy (Zeiss) and Zeiss 710 NLO spectral confocal microscope, and further processed with AxioVision software (Zeiss).

### 4.8 H&E staining and immunohistochemistry

The subrenal capsule transplantations were decalcified with 10% EDTA (pH 7.0) for 2 weeks, then followed by wax sections with a 7‐μm thickness. The sections were rehydrated through graded ethanol and then stained with H&E (catalogue no. C0105, Beyotime, Shanghai, China) as the official protocol from the manufacturer. For immunohistochemistry, anti‐DSPP (1:100, bs‐8557R, Bioss) was used as the primary antibody.

### 4.9 Establishment of EGFP‐mNCCs cell lines

The EGFP sequences were cloned into pSin‐based lentiviral plasmid (Addgene) to construct EGFP vectors. At P2, the cultured cells were transduced using pSin‐based lentiviruses to establish EGFP‐mNCCs. In brief, 4 × 10^4^ normal cells in a six‐well dish were infected overnight with viral supernatants generated by transfection of 293T cells with Lentiviral pSin vectors containing the cDNAs of EGFP. Two rounds of infection were successfully performed. Polybrene (Sigma‐Aldrich) was added to increase infection efficiency. To assess the effect of EGFP expression in the cells, FACS analysis was performed.

### 4.10 Reconstitution of bioengineered tooth germs and subrenal capsule assays

The molar tooth germs were dissected from the mandibles of E14.5 mice and incubated in 0.75 mg/mL Dispase II for 35 min at 37ºC. The dental epithelium was separated from the dental mesenchyme. Meanwhile, cells at P3 or P5 for each further tooth formation experiment were prepared for two purposes: one was collected and lysed with 500 μL TRIzol (Invitrogen) as RNA backup sample for further possible RNA sequencing, and the other was harvested with 10 × 10^5^ cells in per 1.5 mL Eppendorf tube and centrifuged for 4.5 min at 6600 rpm. Cells were aggregated to make pellets by incubation at 37ºC for 2–4 h. The pellets were blown down by 1 mL pipette as the mesenchymal part of the bioengineered tooth germs. A piece of dissected epithelial sheet was placed on the top of the cell pellet. The recombinant explants were cultured with Trowell‐type system in DMEM/HG (high glucose) containing 10% FBS overnight before being transplanted into the renal subcapsular layer of adult ICR mice. Usually, 8–12 recombinant specimens were transplanted into per kidney. After 3 weeks, the host mice were sacrificed to obtain the calcified tissues.

### 4.11 RNA sequence analysis

According to the results of tooth formation, the cell samples were divided into two groups: success and fail groups in tooth formation (T vs. NT), and the RNA backup samples of cells in these two groups were selected. The operation of RNA sequence analysis was the same as the reference.^42^ Briefly, the total RNA was extracted with TRIzol (Invitrogen) according to the manufacturer's instructions; mRNA was then purified and further reverse transcribed, labelled and amplified to generate sequencing‐ready cDNA library with TruSeq RNA Sample Prep Kit (Illumina, USA). The cDNA library concentration was determined with Qubit dsDNA HS Assay kit (Vazyme). The cluster generation and sequencing were performed on Novaseq 6000 S4 platform or NextSeq 500 platform (Illumina).

### 4.12 Statistical analysis

The cell population densities of positive reaction in immunofluorescence are measured in cell counts per unit area, assuming that the cells are 0.6 mm long in the plane normal to the sights. Statistical significance was illustrated as *p* > 0.05 (NS), *p* < 0.05 (*), *p* < 0.01 (**) or *p* < 0.001 (***).

## AUTHOR CONTRIBUTIONS

JC and YW conceived and designed the study. JC and SC designed the experiments. YZ, XL, SC, DW, CL, DY, JZ and YZ performed the experiments. YZ, SC, XC, XL, ZW, BF, DQ, DP, YW and JC analysed the data. YZ, SX, YW and JC wrote the manuscript. All authors commented on the manuscript. YW and JC conceptualised and supervised the project and wrote the manuscript.

## CONFLICT OF INTEREST STATEMENT

The authors declare no conflicts of interest.

## Supporting information


**DATA S1:** Supporting information

## Data Availability

Single‐cell RNA‐Seq, bulk RNA‐seq and metadata supporting the conclusions of this article are available in the NCBI Sequence Read Archive (SRA) database under the BioProject PRJNA832916. The plasmids of hybridisation probe in WISH are available on request to the senior corresponding author (JC).
