# Supplementary material for: Single‐cell RNA‐seq of in vitro expanded cells from cranial neural crest reveals a rare odontogenic sub‐population
Source: Cell Prolif. 2024 Jan 9;57(6):e13598. doi: 10.1111/cpr.13598 (PMC11150137; doi:10.1111/cpr.13598)
Supplement: Supplementary file 1 — DATA S1: Supporting information [file CPR-57-e13598-s001.docx]

**Single-Cell RNA-Seq of *in vitro* Expanded Cells from Cranial Neural Crest Reveals a Rare Odontogenic Subpopulation**

Yifan Zhao^1,2,3,4, †^, Shubin Chen^1,3,4,†^, Xiaobo Liu^5,†^, Xiaoming Chen^4,6^, Dandan Yang^7^, Jiashu Zhang^8^, Di Wu^3,4^, Yanmei Zhang^4^, Si Xie^1,2^, Xiaomei Li^1^, Zhiyuan Wang^8^, Bo Feng^9^, Dajiang Qin^1,2,3^, Duanqing Pei^2,10^, Yaofeng Wang^1,2,*^, Jinglei Cai^1, 2, 4, 11,12*^

*^1^ Innovation Centre for Advanced Interdisciplinary Medicine, The Fifth Affiliated Hospital of Guangzhou Medical University, Guangzhou, 510799, China.*

*^2^ Centre for Regenerative Medicine and Health, Hong Kong Institute of Science & Innovation, Chinese Academy of Sciences, Hong Kong SAR, China.*

*^3^ Bioland Laboratory, Guangzhou Regenerative Medicine and Health Guangdong Laboratory, Guangzhou, 510005, China.*

*^4^ CAS Key Laboratory of Regenerative Biology, Guangdong Provincial Key Laboratory of Stem Cell and Regenerative Medicine，Guangzhou Institutes of Biomedicine and Health, Chinese Academy of Sciences, Guangzhou, 510530, China.*

*^5^ Laboratory of Cancer Precision Medicine, the First Hospital of Jilin University, Changchun, 130061, China.*

*^6^ Guangdong Provincial People's Hospital Ganzhou Hospital, Ganzhou Municipal Hospital, Ganzhou, 341099, China.*

*^7^ Experimental Center of Pathogenobiology Immunology, Cytobiology and Genetics, Basic Medical College, Jilin University, Changchun, 130021, China.*

*^8^ Innovation centre for Translational Medicine, The Fifth Affiliated Hospital of Guangzhou Medical University, Guangzhou, 510530, China.*

*^9.^ School of Biomedical Sciences, The Chinese University of Hong Kong, Hong Kong SAR, China.*

*^10^ Laboratory of Cell Fate Control, School of Life Sciences, Westlake University, Hangzhou, 310024, China.*

*^11^ Guangzhou Key Laboratory of Enhanced Recovery after Abdominal Surgery，The Fifth Affiliated Hospital of Guangzhou Medical University, Guangzhou, 510530, China.*

*^12^ Institute for Stem Cell and Regeneration, Chinese Academy of Sciences, Beijing 100101, China.*

^†^ These authors contributed equally.

* Correspondences: caijinglei@gzhmu.edu.cn (J.C.), yaofeng.wang@hkisi-cas.org.hk (Y.W.)

**Supplementary Information: Figures**

**
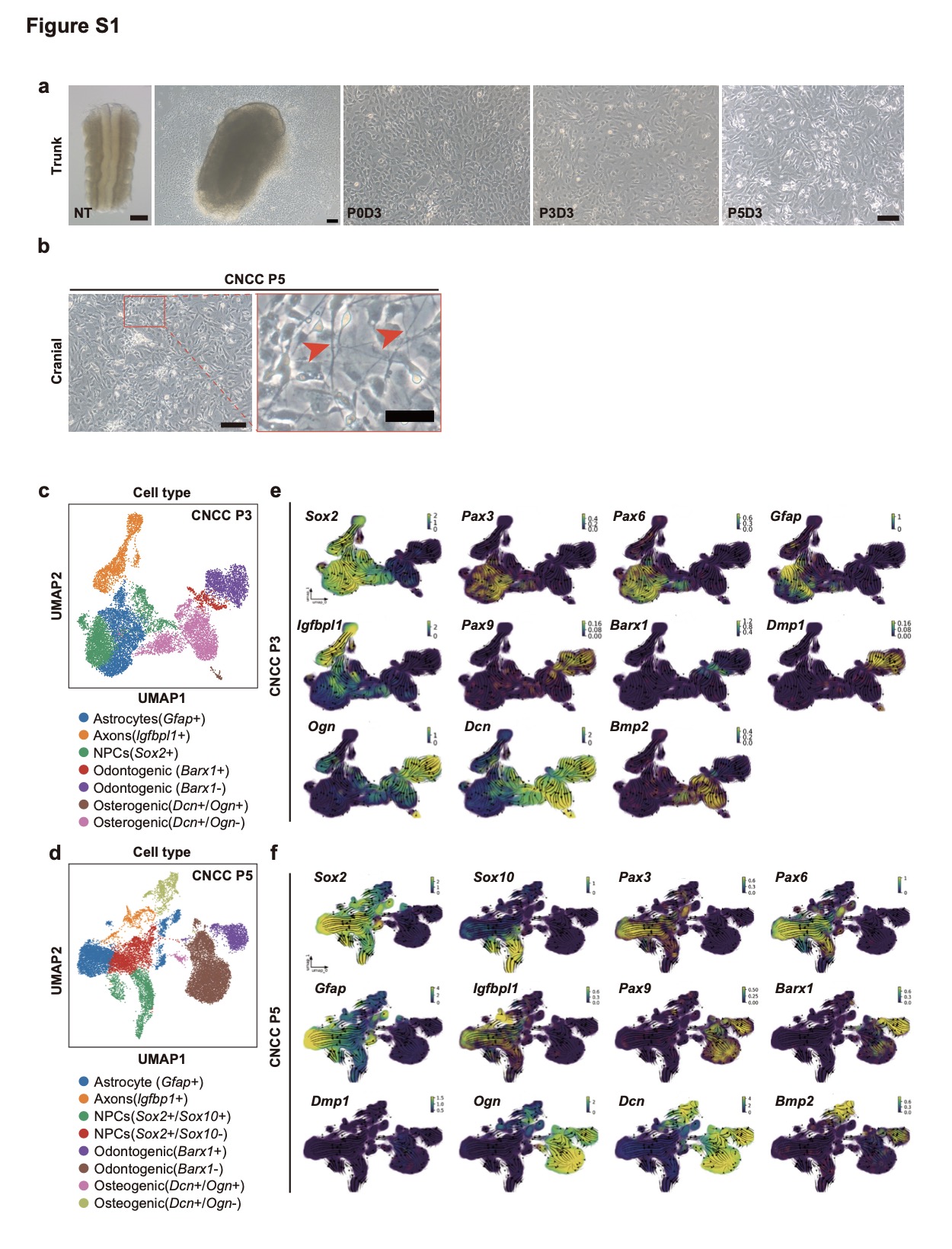
**

**Figure S1**

(a) Flow chart of TNCCs isolation and culture. Scale bar corresponds to 100 μm.

(b) Enlarged schematic diagram of cell morphology and nerve filament. Scale bar: 100 μm, 50 μm.

(c) UMAP visualization of cells colored by cell-type assignment of P3 cells.

(d) UMAP visualization of cells colored by cell-type assignment of P5 cells.

(e) Gene expression patterns of important genes at P3 as UMAP visualization

(f) Gene expression patterns of important genes at P5 as UMAP visualization


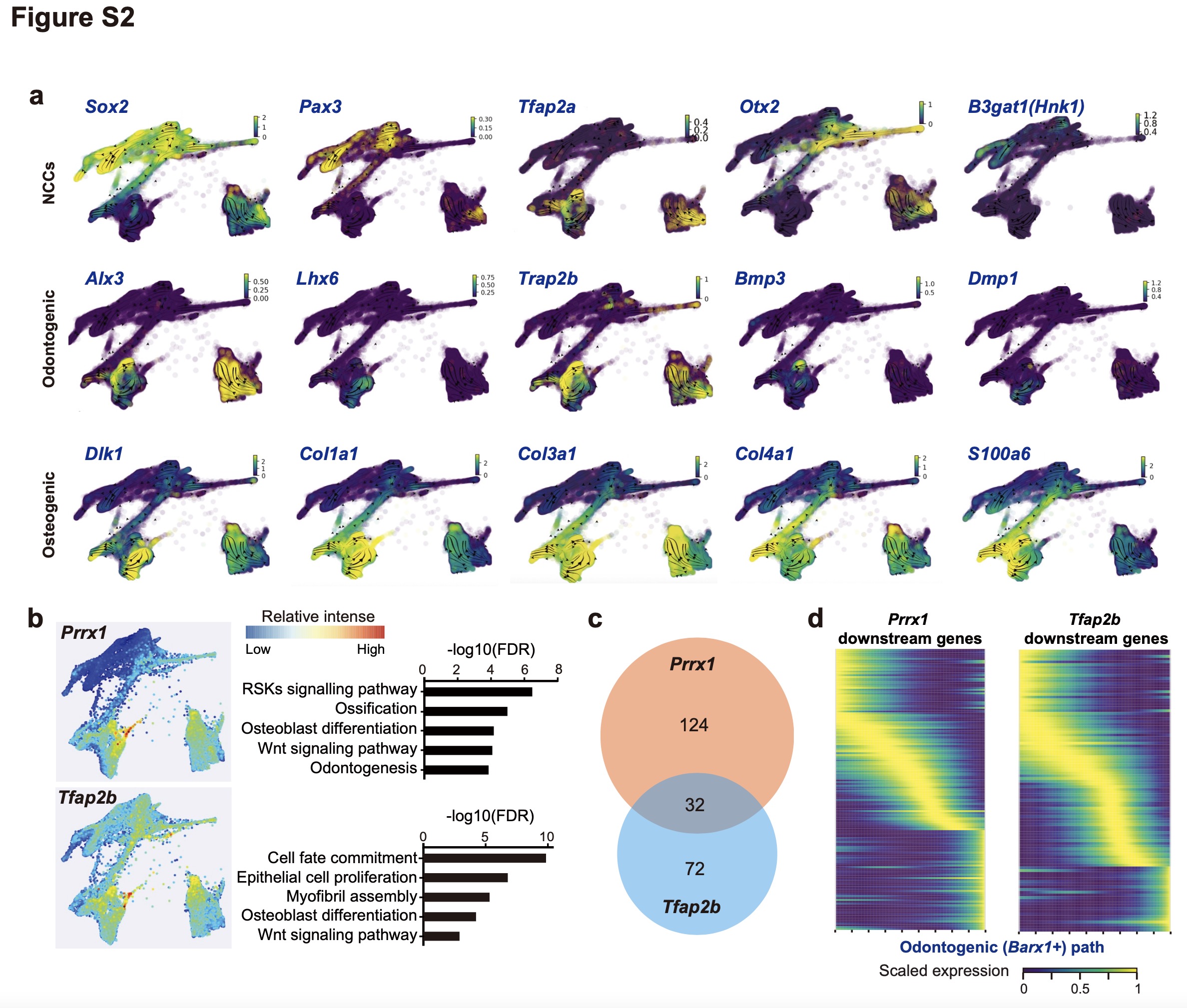


**Figure S2**

1. Gene expression patterns of NCCs markers (top), odontogenic markers (middle), and osteogenic markers in the cell landscape of i*n vitro* cultured CNCCs.
2. SCENIC analysis of *Prrx1*-GRN (top) and *Tfap1b*-GRN (bottom) as the key gene networks to drive the odontogenic differentiations: Area under the curve (AUC) levels of GRN in cell landscape of i*n vitro* cultured CNCCs (left) and gene ontology analysis of genes in the networks (right).
3. Venn diagram of overlapping genes in *Prrx1*-GRN and *Tfap1b*-GRN networks.
4. Heat map showing the downstream genes of *Prrx1*-GRN (left) and *Tfap1b*-GRN (right) expressed in *Barx1+* odontogenic path across the pseudotime.
